# Electron-Energy Dependent Excitation and Directional Far-Field Radiation of Resonant Mie Modes in Single Si Nanospheres

**DOI:** 10.1021/acsphotonics.5c00173

**Published:** 2025-07-24

**Authors:** Théo Soler, Evelijn Akerboom, P. Elli Stamatopoulou, Hiroshi Sugimoto, Minoru Fujii, Saskia Fiedler, Albert Polman

**Affiliations:** † Center for Nanophotonics, 55952 NWO-Institute AMOLF, Science Park 104, 1098 XG Amsterdam, The Netherlands; ‡ Institute of Nanotechnology, 150232 Karlsruhe Institute of Technology, Kaiserstr. 12, 76131 Karlsruhe, Germany; § Department of Electrical and Electronic Engineering, Graduate School of Engineering, 12885 Kobe University, Rokkodai, Nada, Kobe 657-8501, Japan

**Keywords:** Mie resonances, Kerker condition, dielectric, cathodoluminescence spectroscopy, directionality, electron microscopy, angle-resolved

## Abstract

High-energy electron beams with energies in the 15–30 keV range are used to excite optical Mie modes in crystalline Si nanospheres with radius 80–100 nm. Cathodoluminescence (CL) spectra show emission from resonant electric and magnetic dipole and quadrupole modes, with relative intensities that depend strongly on electron energy and impact parameter. The measured trends are explained by a coupling model in which the electron-energy dependent CL excitation probability–and thus the CL emission–is proportional to the Fourier transform of the modal electric field at a spatial frequency determined by the electron velocity. As a result, the coupling to a specific resonant mode is strongly dependent on the electron energy and the impact parameter of the electron beam. This enables the selective enhancement of CL emission from a resonant mode by phase-matching with the electron velocity. A systematic study of spatial excitation probability for the electric dipole mode as a function of electron energy further confirms the validity of the coupling model. Angle-resolved cathodoluminescence measurements show strong directional emission due to far-field interference of coherently excited Mie modes. By varying the electron energy and impact parameter the intensity and interference of these modes can be controlled and the angular distribution tailored. The insights in the localized deep-subwavelength coherent excitation of resonant Mie modes explored here are important for studies in light-emitting nanostructures, sensors, and photovoltaics, in which the interplay of local modes and far-field directional emission must be controlled.

## Graphical abstract

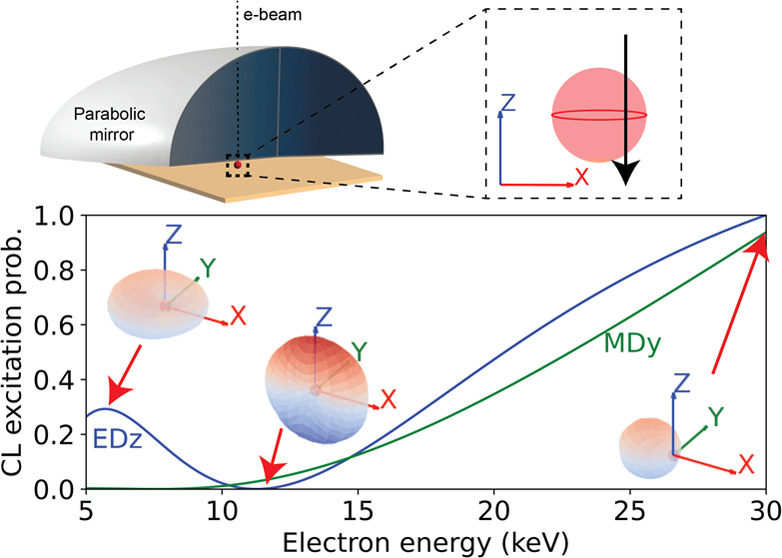

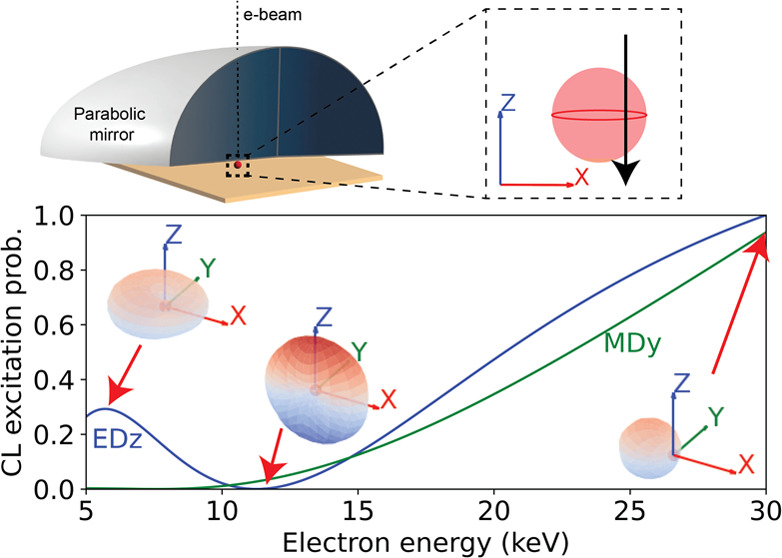

## Introduction

Controlling light in photonic nanostructures at a deep-subwavelength scale is crucial for many technological advancements, ranging from quantum technologies to solar applications.^1^ In particular, directional light emission from photonic structures is important for optimizing the performance of light-emitting diodes and lasers, sensors, and photovoltaics.^2,3^ As previously shown, selective coupling to resonant optical nanoantennas provides a unique means to tailor the directionality of optical emitters.^4,5^ Both metallic and high-index dielectric nanostructures are of interest for these applications. While the former can support plasmonic resonances which are mainly governed by the material properties, high-index dielectric nanostructures are particularly interesting since they can support a wide range of Mie resonances that are electric and magnetic in nature with very low absorption losses (much lower than for plasmons) and high *Q*-factor (narrow emission line width).^3,6–8^ Previous works have investigated the resonances of Si nanostructures due their ability to support dipolar and quadrupolar Mie modes in the visible spectral range where Si shows relatively low loss due to its indirect electronic bandgap.^9,10^ This allows for strong tunability of light-matter interactions by tailoring the size, shape and dielectric environment of the Mie particles, thereby controlling the resonant energy and angular emission distribution of the electric and magnetic multipolar modes. Furthermore, it has been shown that the interference of these multipolar modes can lead to light emission in specific directions, similar to the Kerker effect in Mie theory.^11^ In order to take advantage of these properties, it is crucial to control and probe the electric fields at the nanoscale.

To study optical near fields of nanostructures at a spatial resolution below the optical diffraction limit, three different electron microscopy techniques have been developed in recent years: cathodoluminescence (CL) spectroscopy, electron energy loss spectroscopy (EELS) and, more recently, photon-induced near-field electron microscopy (PINEM). In all cases the electron is used to probe the electric field along the electron trajectory that is either induced by the electron itself (for CL and EELS) or by an external laser (for PINEM). In the case of CL and EELS, a bypassing electron polarizes the material, creating an electric near field that acts back on the electron, causing it to lose energy. The total energy loss is measured in EELS, while the radiating part can be detected in the far field as CL.^12^ The CL emission from plasmonic or Mie particles has a coherent phase relation with the electron impact.^13^ The advantage of CL spectroscopy is its capability to probe the electric field with nanometer precision due to the small focus dimension of the electron beam.^14^ The femtosecond electromagnetic field oscillation created by a bypassing electron offers a broadband source of modal excitation, enabling broad spectral analysis in a single CL measurement.^15^

In nanophotonics, CL and EELS have mostly been used to study plasmonic nanostructures.^12,16–18^ Recently, dielectric structures have also been investigated using electron beams. Coenen et al. characterized higher-order electric and magnetic Mie resonances in lithographically fabricated Si nanodisks using CL in a scanning electron microscope (SEM).^6^ Matsukata et al. experimentally demonstrated CL spectra of resonant modes in Si nanospheres in a transmission electron microscope (TEM) and observed strong directionality in the CL radiation.^19^ And recently, Fielder et al. explored the interplay of Mie resonances and transition radiation in CL spectra of Si nanospheres.^20^ These works show the potential of CL spectroscopy to investigate and control directional light emission. However, so far, the relation between the strength of the electron-mode coupling, the spectrum and the directionality of the CL emission in dielectric nanostructures has not been studied in a comprehensive way.

In this work we demonstrate that we can achieve directionality of the emission from electron-excited dielectric resonators, showing the electron beam analogue to the Kerker effect that is known from optics. It also enables us to sensitively test the model for electron-mode coupling. We investigate the coupling of the electron beam to specific resonances in individual Si nanospheres both theoretically and experimentally. We take advantage of the fact that, at a given velocity, the electron resonantly couples to specific spatial frequency components of the oscillating electric near-field distribution in the particle.^21^ By varying the electron energy and impact parameter in an SEM we selectively couple to modes with specific spatial near field distributions. We then control the interference of the excited modes to create specific far-field radiation patterns. We take advantage of the spherical symmetry to exploit analytical solutions for the mode profiles from Mie theory. Our results provide fundamental understanding of electron-mode coupling and provide a framework to control and design directional light emission from single dielectric nanostructures.

## Theory

Fundamentally, the electron energy loss that leads to CL is determined by the coupling between the electron and the electric near-field component in the direction along the electron trajectory. The CL and EELS spectral intensities are then proportional to the work done by this field component on the electron. In the nonrecoil approximation, i.e., assuming that the electron velocity remains constant when traversing the nanostructure, the CL emission intensity (Γ_CL_) for an electron traveling along the *z*-axis for a particle with a single resonant mode, is proportional to^12,22^

1 $$\Gamma_{CL}\left(R,\omega \right)\propto {\left|\int dz\;E_{z}\left(R,z,\omega \right)e^{-i\left(\omega /v\right)z}\right|}^{2}$$

with ***R*** = (*x*,*y*) the impact parameter in Cartesian coordinates, ω the angular frequency (ω = 2π*c*/λ), *E*_*z*_ the *z*-component of the induced electric field, and *v* the electron speed. Classically, eq 1 can be interpreted as the net energy loss along the electron trajectory: the integrated effect of the acceleration and deceleration of the electron caused by its interaction with the induced oscillating electric near field. The resulting absolute CL intensity is then determined by this energy loss and the nanostructure’s albedo, given by the relative contribution of radiative and nonradiative losses. Equation 1 shows that the electron beam effectively takes the Fourier transform of optical near fields at a spatial frequency (*q* = ω/*v*): the observed coupling strength (CL intensity) at a given electron velocity then directly represents the strength of the spatial frequency *q* in the distribution of the near fields in the *z*-direction. The CL emission at a given impact parameter is maximized when the electron motion is phase-matched with the induced oscillating fields for that impact parameter. For a *z*-oriented dipole mode excited in the center–characterized by sharp features at the edge of the particle–the phase matching condition is *q* ∼ (2*n* + 1)­π/*D*, with *D* the diameter of the particle and *n* an integer.^21^

First, we theoretically study the configuration where an electron beam couples to the *z*-oriented electric dipole (ED) in a Si sphere with radius *r* = 100 nm. In Figure 1a, we show the real part of the *z*-component of the electric field incident at such a particle at λ = 600 nm. The induced electric fields are calculated using classical Mie theory^23^ using optical constants for crystalline Si from Green et al.^24^ Next, we study the CL emission for two electron beam impact parameters in spherical coordinates (*b*), given by the distance to the center of the particle; (1) electrons passing through the particle center (*b* = 0 nm) and (2) near the outer edge (*b* = 90 nm). Figure 1b shows the real part of the *z*-component of the induced electric field along the electron trajectory for the corresponding two impact parameters. By taking the Fourier transform of the *E*_*z*_ profiles, we calculate the CL emission probability associated with this mode for an electron energy in the range between 5 and 30 keV (Figure 1c). For electrons impacting in the center of the particle, we find a maximum CL excitation probability at 12 keV, corresponding to a spatial frequency of 0.047 nm^–1^. Here, the electron is in phase with the excited mode (ED) causing maximal electron energy loss and hence, maximum CL emission. This maximum CL excitation probability corresponds to the second-order phase-matching condition for center excitation, *q* = 3π/*D*. The first peak at lower *q* (higher electron energy) is found at 0.016 nm^–1^ (165 keV). For an electron beam exciting the nanosphere on the edge, no clear CL excitation probability maximum is observed in the studied range and the CL emission increases with electron energy. The analysis in this section clearly shows that the electron beam coupling to dielectric Mie modes depends strongly on the impact parameter and electron velocity. As we will show next, this provides a unique means to tailor the coupling of the electron to specific resonant modes, thereby controlling both the spectrum and angular distribution of the CL emission.

**Figure 1:**
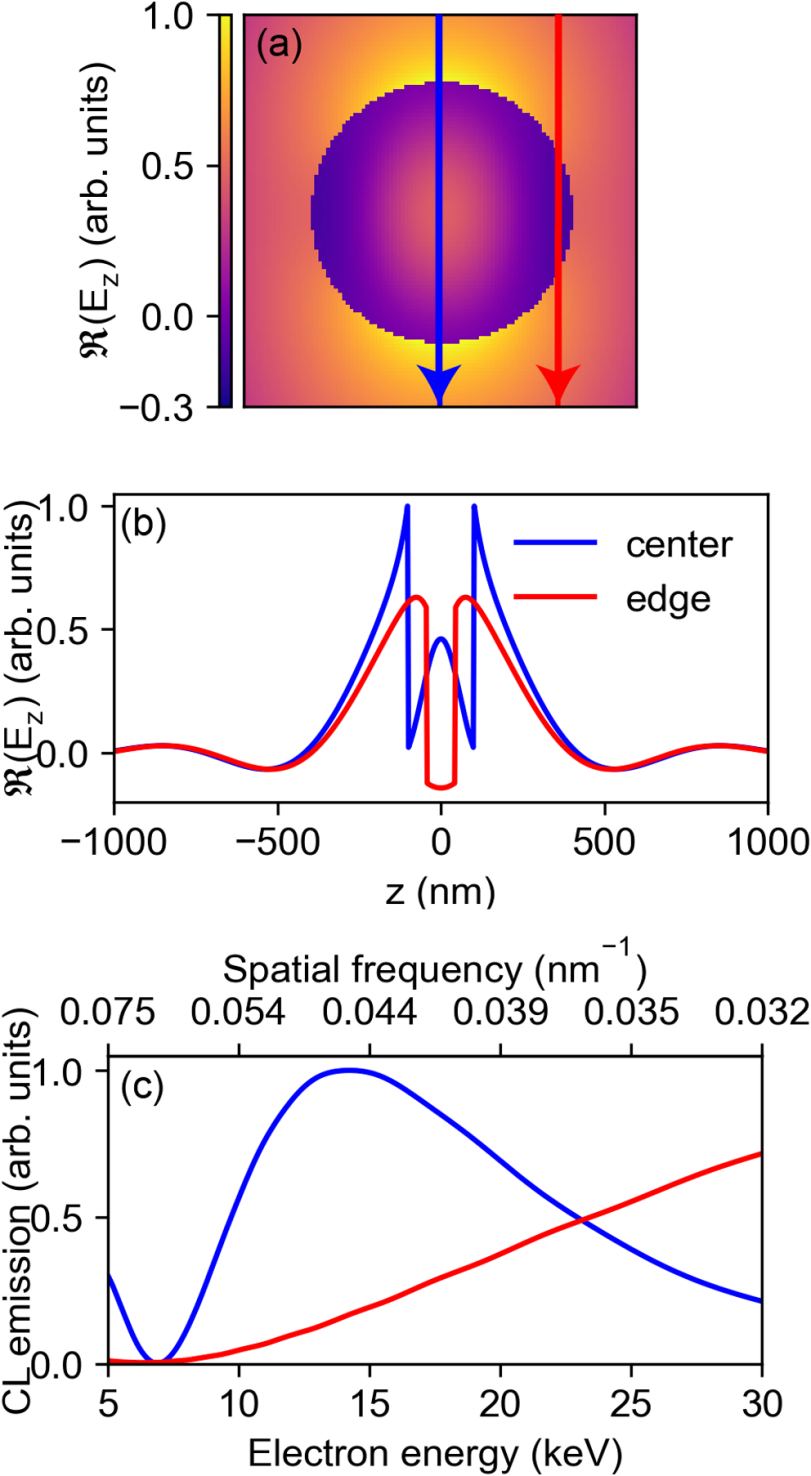
(a) Real part of the *z*-component of the electric field of a *z*-oriented electric dipole mode induced with a plane wave polarized along *z* at λ = 600 nm in a dielectric nanosphere with a radius *r* = 100 nm. (b) Real part of the *z*-component of the electric field along the electron trajectory for two electron beam impact parameters; one at *b* = 0 (blue) and the other at *b* = 90 nm (red), indicated by the arrows in (a). (c) Calculated normalized CL emission intensity as a function of electron energy using eq 1. The CL emission intensity is proportional to the square modulus of the spatial Fourier transform of the *z*-component of the electric field for the two electron trajectories of panel (b). The top axis shows the corresponding spatial frequency (*q* = ω/*v*) for λ = 600 nm.

While the analysis above provides basic insight into the coupling concept, in experiments the modal field distributions depend on the electron energy and impact parameter and, due to the coherent excitation process, create modal interference in the far field. In the remainder of this work, we use the electrodynamical electron-mode coupling model to calculate the CL excitation and emission spectra and far-field angular distributions.^25^ This model provides an analytical derivation for the electric and magnetic fields in the case of coherent excitation of multiple modes by an electron beam crossing the particle (see Methods section). The resulting expression for the CL emission probability is a sum over the electric and magnetic modes for orders l and orientation with index *m*, given by

2 $$\Gamma_{\mathrm{CL}}\left(\omega \right)\propto \sum_{l=1}^{\infty }\sum_{m=-l}^{+l}\left\{{\left|b_{lm}^{\mathrm{II}}\right|}^{2}+{\left|a_{lm}^{\mathrm{II}}\right|}^{2}\right\}$$

where $b_{lm}^{\mathrm{II}}$ denote the magnetic and $a_{lm}^{\mathrm{II}}$ the electric modes of the scattered electromagnetic field in the far field. Mode indices refer to the mode order– l=1 for magnetic or electric dipole modes (MD, ED), l=2 for electric and magnetic quadrupole modes (EQ, MQ)–and orientation (for an out-of-plane dipole, *m* = 0, and for an in-plane dipole, *m* = ±1).

### Experiments

Single-crystalline nanospheres with radii between 80 and 100 nm were fabricated using the method developed by Sugimoto et al.^26^ and deposited on a 15 nm-thin Si_3_N_4_ TEM membrane (see Methods section). CL experiments were performed using a SEM operating at acceleration voltages in the range of 15–30 keV. A half-parabolic mirror placed between the sample and the electron column collects the emitted CL and directs it to a spectrometer for spectral analysis. Angular CL emission distributions were recorded in Fourier mode by projecting the emission on a CCD imaging sensor, using a 50 nm wide bandpass filter (see Methods section).

## Results and Discussion

### Spectral Cathodoluminescence

First, we study the CL emission for a Si nanosphere with a radius of 96 nm, excited by a 30 keV electron beam. Figure 2a shows the measured and calculated CL spectrum at *b* = 86 nm using eq 2. The experimental spectrum is corrected considering the collection efficiency of the parabolic mirror and the spectrometer. In the spectra, we find four clear peaks, corresponding to the different modes supported by the Si nanosphere. Experimentally, we find peaks corresponding to MD at λ = 744 nm, ED at λ = 605 nm, MQ at λ = 563 nm, and EQ at λ = 487 nm. For all experimental resonances, the peak energy is red-shifted compared to the theoretical ones. We ascribe this to the effect of mode coupling to the Si_3_N_4_ substrate.^27,28^ We observe good agreement between experiment and theory for the trend in line width of the modes, where quadrupolar modes exhibit narrower resonances compared to the broader dipolar modes. The modal intensities for ED and MD modes show a similar trend for experiment and theory, while an opposite trend is observed for the EQ and MQ modes. The experimental EQ mode is more intense than the MQ one, while theory shows the opposite. We primarily attribute this discrepancy to the increase of the absorption coefficient of Si below λ = 500 nm due to the direct bandgap at 450 nm, which strongly reduces the calculated intensity at that wavelength.

**Figure 2:**
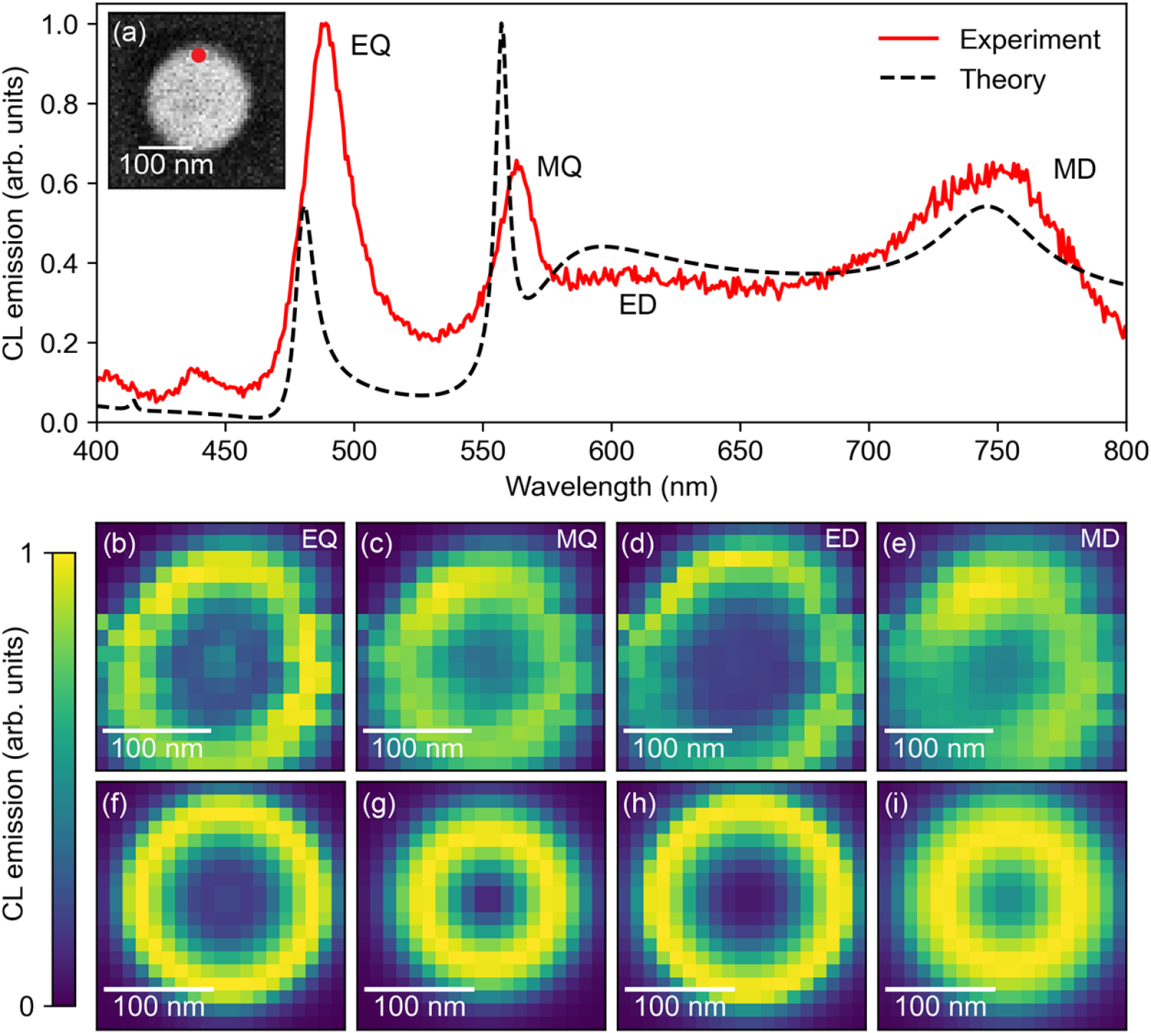
(a) Measured (red) and calculated (dashed black) CL spectra from a *r* = 96 nm Si nanosphere excited with 30 keV electrons. The measured spectrum is taken at *b* = 86 ± 5 nm, while *b* = 86 nm is used for the calculated spectrum. The inset shows the SE image of the Si nanosphere with the red dot indicating the position of the electron beam excitation for the experimental spectrum (*b* = 86 ± 5 nm). (b–e) Measured and (f–i) calculated CL maps at the resonance wavelengths of the different multipoles. The measured CL maps are averaged over a bandwidth of 4 nm with the center wavelength of 487 nm (EQ), 563 nm (MQ), 605 nm (ED), and 744 nm (MD). The theoretical CL maps are calculated using eq 2 considering all modes until $l_{\max}=2$ and for wavelengths of 450, 557, 595, and 745 nm, respectively. Pixel sizes of 14 nm for the experimental and 12 nm for the theoretical CL maps are used, respectively.

In Figure 2b–i, we display the measured spatial distribution of the CL intensity for the same nanosphere for each of the Mie modes in (a). We show the CL intensity at the center wavelength of the four resonances. The experimental data (b–e) are averaged over a 4 nm-bandwidth at λ = 487 nm (EQ), λ = 563 nm (MQ), λ = 605 nm (ED), and λ = 744 nm (MD). The corresponding theoretical data (f–i) are calculated at the peak wavelengths of 450, 557, 595, and 745 nm, for EQ, MQ, ED, and MD, respectively. We note that CL analysis is an excitation spectroscopy technique in which the electron excitation is spatially well-defined and the spatial resolution in the CL excitation maps is determined by the electron trajectory. The CL emission is collected from the entire particle within the focal collection area and numerical aperture of the parabolic mirror. We further note that the resolution of the secondary electron (SE) image shown in the inset of Figure 2a is much higher than the CL resolution. In contrast to the electron trajectory-dependence in CL, SEs mainly originate from the surface of the sample resulting in an estimated spatial resolution of 1–2 nm. This allows us to accurately determine the radius of the Si nanosphere with a simple postprocessing step.

The CL maps in Figure 2 clearly show that electron beam excitation results in characteristic spatial excitation distributions for the different Mie modes in the Si nanosphere, similar to what was found before.^19,29^ Next, we analyze these data using the coherent coupling model described above. Many trends in the measured maps match well with the calculated CL emission maps. For all modes, the excitation efficiency is highest at the edge of the particle, in both experiment and theory. Small features such as the larger area of reduced intensity in the center for the ED mode are also observed theoretically and experimentally.

The theoretical ED and EQ modes show higher overall intensities at the edges, compared to MD and MQ mode; an effect that is also visible in the measurements. We note that the sharply concentrated ring in the experiment of Figure 2d directly shows the high lateral spatial resolution of the CL measurement. From a comparison with the theoretical map, we derive that the spatial resolution is better than the pixel size in these experiments, i.e., below 14 nm. This is a result of the electron beam focal diameter on impact (5–10 nm) and small straggle of the electron beam due to inelastic scattering, which is in qualitative agreement with Monte Carlo simulations of electron trajectories for the studied geometry.

The nonspherical shape of the CL maps is attributed to small sample drift during each of the measurements. Further asymmetries in the measurements are due to the asymmetry of the parabolic mirror light collection geometry, which makes the CL collection efficiency for each mode dependent on its angular emission distribution. This effect is visible in the CL maps for the ED and MD modes, for which the bottom left corner is slightly dimmer: CL generated in that area beams toward the mirror’s opening and is therefore only partly collected. These results already hint at the specific angular emission characteristics of electron-excited Mie modes that we will study in detail further on.

The good agreement between experiment and theory now allows us to study the effect of electron energy on the electron-mode coupling and assess the spatial frequency formalism for resonant modal excitation as described above. To do so, we study the dependence of CL emission on the electron energy ranging from 15 to 30 keV corresponding to a spatial frequency range of 0.044–0.032 nm^–1^ at the ED resonance wavelength (for electron-energy dependent spectra, see Figure S1). Figure 3 shows the experimental (a) and theoretical (b) CL maps as a function of electron energy and impact parameter along the center line of the sphere (crosscut through the nanosphere from left to right) for the ED resonance (λ = 605 ± 2 nm for experiments and λ = 595 nm for theory). The electron-energy dependency of the other Mie resonances is shown in Figure S3. The main trend of the measurements follows the calculated data: the excitation efficiency shows a minimum value which is strongly dependent on the electron energy. This clearly underlines the phase-matching condition for the ED: the minimal coupling strength is expected at *q* ∼ 4π/D*, with D* the height of the particle at a certain impact parameter, which is visualized by the white dashed lines in Figure 3a,b. In fact, these electron-energy dependent CL measurements allow us to determine the thickness of the nanosphere at different impact parameters using the well-defined phase-matching condition of the ED resonance. Furthermore, for excitation at the edge of the nanosphere, the model predicts a decrease in CL intensity with decreasing electron energy; this is also found in the experimental CL maps, where the intensity at the edge vanishes at the lowest experimentally feasible energy of 15 keV. This originates from the lower CL excitation probability of both the in-plane (*x*-, and *y*-oriented) and the out-of-plane ED modes (z-oriented) near the edge of the particle (cf. blue line in Figure 1c).

**Figure 3:**
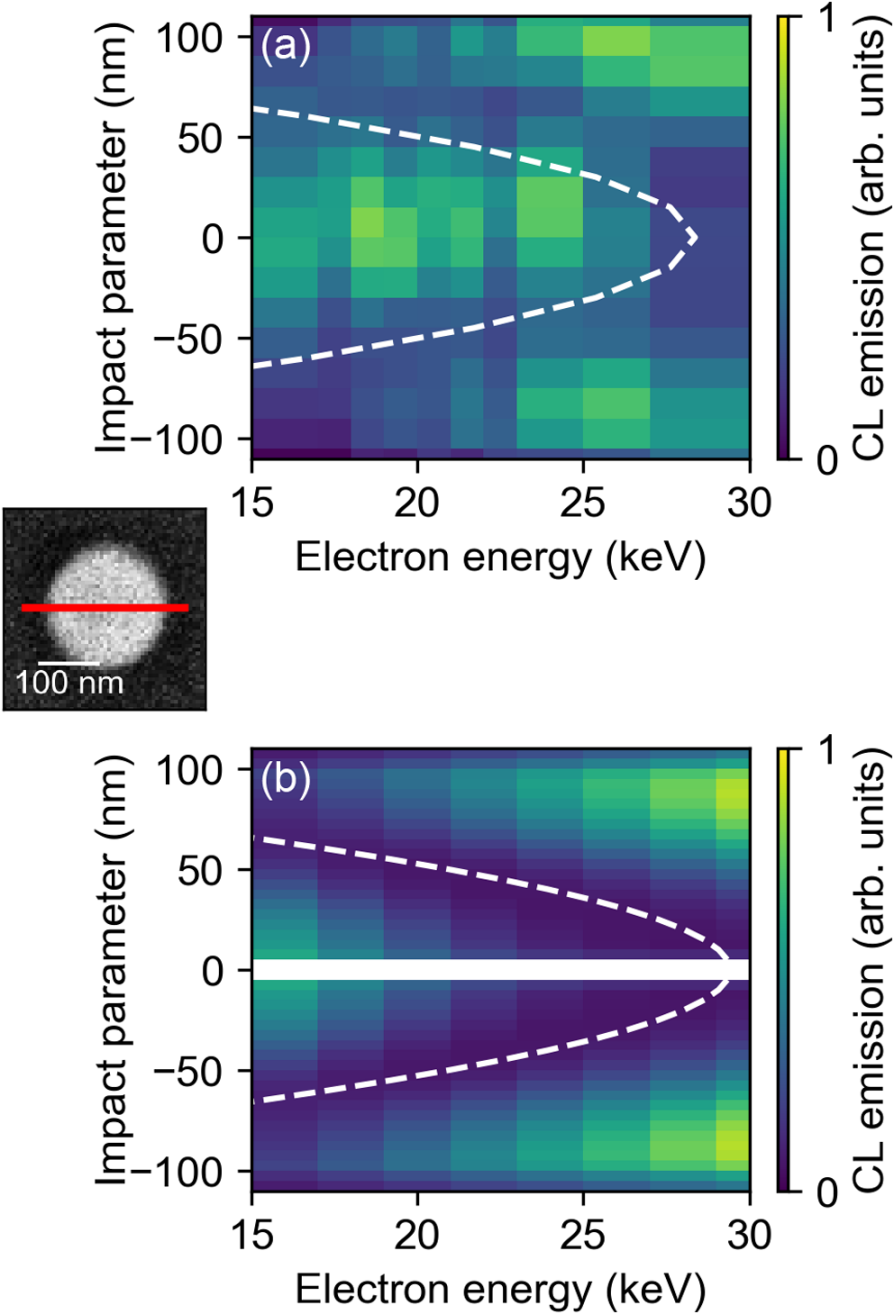
(a) Measured and (b) calculated CL maps obtained by a crosscut through the center line of a Si nanosphere of r = 96 nm excited with electron energies ranging from 15 to 30 keV. The experimental data are collected at a center wavelength of 605 nm with a 4 nm bandwidth. The theoretical calculations are performed at the ED resonance wavelength, λ = 595 nm, using eq 2 considering all modes until $l_{\max}=2$ . The white dashed line in experiments and theory shows the rule-of-thumb for the expected minima of the phase-matching condition for the ED, *q* ∼ 2*nπ*/D*, for *n* = 2 and with D* the height of the particle at a certain impact parameter (for a spherical particle with radius *R*, the height at a certain impact parameter is given by $D^{*}=2\sqrt{R^{2}-b^{2}}$ ).

### Directional Far-Field Emission

With the detailed understanding of selective mode coupling obtained in the previous section, we now study the angular distribution of the CL emission from an individual Si nanosphere and explore how it can be controlled by electron energy and positioning of electron beam. This gives the possibility to control their interference in the far field, thereby creating directionality of CL emission.

First, we theoretically study the angular emission profile for a Si nanosphere (*r* = 96 nm) excited by a 30 keV electron beam, as depicted schematically in Figure 4a. We choose an impact parameter of *b* = 86 nm as we know that close to the edge, the electron can couple to both magnetic and electric modes at this electron energy. The calculated CL spectra is shown in Figure S4, decomposed into the different modes. To achieve directionality, we exploit the Kerker effect known for dielectric spheres. We study the angular emission distribution at two emission wavelengths where the interference of the modes creates directional emission: (1) λ = 760 nm, using the interference between the ED and the MD mode (Figure 4b–e), and (2) λ = 595 nm, using the ED and the EQ mode (Figure 4f–h). The data are derived by calculating the Poynting vector for the coherent sum in the far field of the indicated modes. The full modal solution is obtained by summing all relevant modes with $l_{\max}=2$ .

**Figure 4:**
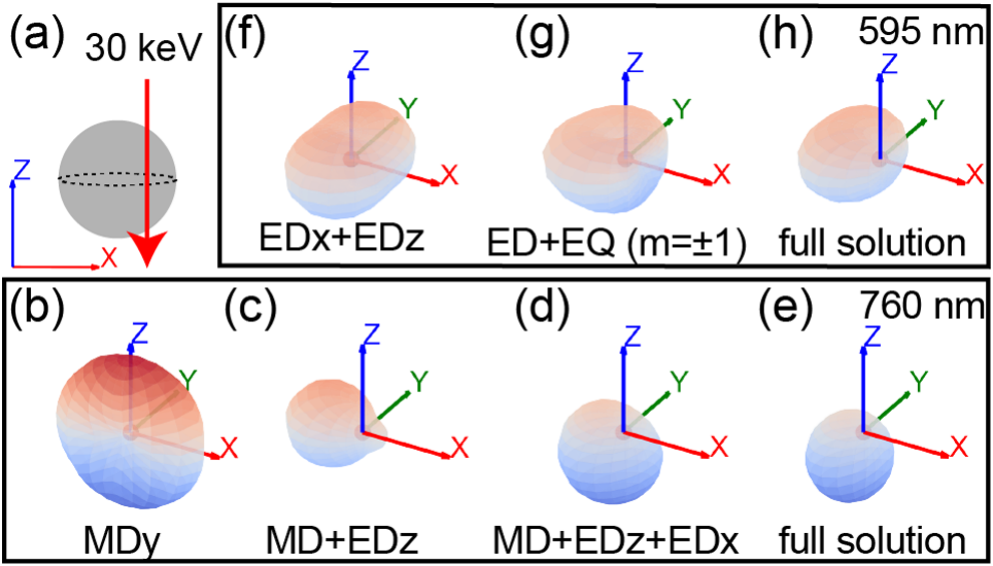
(a) Schematic of excitation configuration: a Si nanosphere with *r* = 96 nm is excited by a 30 keV electron beam at *b* = 86 nm.i (b–h) Far-field radiation patterns at two emission wavelengths: (b–e) λ = 760 nm (MD and ED resonances) and (f–h) λ = 595 nm (ED and EQ resonances). The radiation patterns are calculated by taking the Poynting vector (*S*) of the scattered electromagnetic field of eq 4, coherently summing over the modes. For the full solution, the sum of all modes using $l_{\max}=2$ is considered. The color map visualizes the *z*-direction of the radiation (*S*_*z*_): red for upward and blue for downward intensities.

In Figure 4b we recognize the typical radiation pattern for an MD mode with dipole moment along the *y* direction (MD*_y_*). We note that the electron beam does not couple to the MD*_x_* and MD*_z_* modes because their electric field vectors are perpendicular to the electron beam trajectory.^25^ When adding the contribution of the ED*_z_* mode (Figure 4c), we observe directional CL emission opposite to the electron trajectory. This corresponds to the transverse Kerker effect that has been theoretically studied^30^ and experimentally observed.^31^ When we add the ED*_x_* mode as displayed in Figure 4d, the combined effect with the MD gives a Kerker-type interference along the *z*-axis, creating downward directionality. In the full solution (Figure 4e), adding up all modes with $l_{\max}=2$ , we observe both horizontal and vertical directionality: the CL radiation is directed toward negative *x* and negative *z*. This is the result of the interference of the MD + ED*_z_* mode and of the MD + ED*_x_* mode, respectively. We note that the vertical directionality is downward at wavelengths above the MD resonance wavelength (λ_MD_ = 726 nm) because the ED and MD resonance are in-phase.^31^

In Figure 4f–h, we follow the same steps for the ED resonance at λ = 595 nm. We start by only considering the contributions of the ED modes, namely ED*_z_* and ED*_x_*, resulting in a symmetric emission distribution around the *y*-axis (Figure 4f). Next, in Figure 4g the contribution of the EQ is added, which leads to directionality toward the opposite side of the electron beam impact position. This is due to the interference of the EQ and the ED modes. Comparing this to the full solution shown in Figure 4h, we see that this interference is the largest contribution to the full solution.

This theoretical study shows that controlling the interference of coherently excited Si Mie modes creates strong directional CL emission in the far field, both in the horizontal and vertical direction. For emission at *λ* = 760 nm there are two dominant mechanisms: interference of the MD mode with the out-of-plane ED mode (ED*_z_*) resulting in horizontal directionality, and interference of the MD mode and the in-plane ED mode (ED*_x_*) resulting in vertical directionality. At *λ* = 595 nm, the coherent addition of the EQ mode is necessary to generate horizontal directionality, due to the absence of the MD mode.

With this theoretical framework for directionality, we now experimentally exploit this formalism for the selective excitation of resonant modes thereby controlling their interference and thus tailoring the angular emission distribution. In these experiments, in which we use a slightly smaller nanosphere (*r* = 85 nm), we vary both the electron energy and the impact parameter.

Figure 5a shows the CL excitation probability as a function of electron energy at impact parameter *b* = 42 nm calculated for specific modes using eq 2. We consider the CL emission at *λ* = 585 nm, in between the ED and the MD resonance wavelength. We find a strong dependence of the CL excitation probability for specific modes on the electron energy. For example, at 15 keV the electron does not couple to the ED*_z_* mode but excites only the ED*_x_* mode, while at 30 keV the contribution of the ED*_z_* mode is strongest. Using these CL excitation probabilities, we calculate the angular CL emission profile at two electron beam energies. At 30 keV (Figure 5c) the theory predicts a strong horizontal directionality toward the opposite side of the excitation (the negative *x*-direction), originating from Kerker-like interferences of ED*_z_* + MD and ED*_z_* + EQ modes. This strong directionality reverses to the opposite direction, and decreases in strength, for excitation with a 17 keV electron beam (Figure 5b). This comparison shows the strong effect of far-field interference of the coherently excited Mie modes in Si nanospheres on the angular radiation profile.

**Figure 5:**
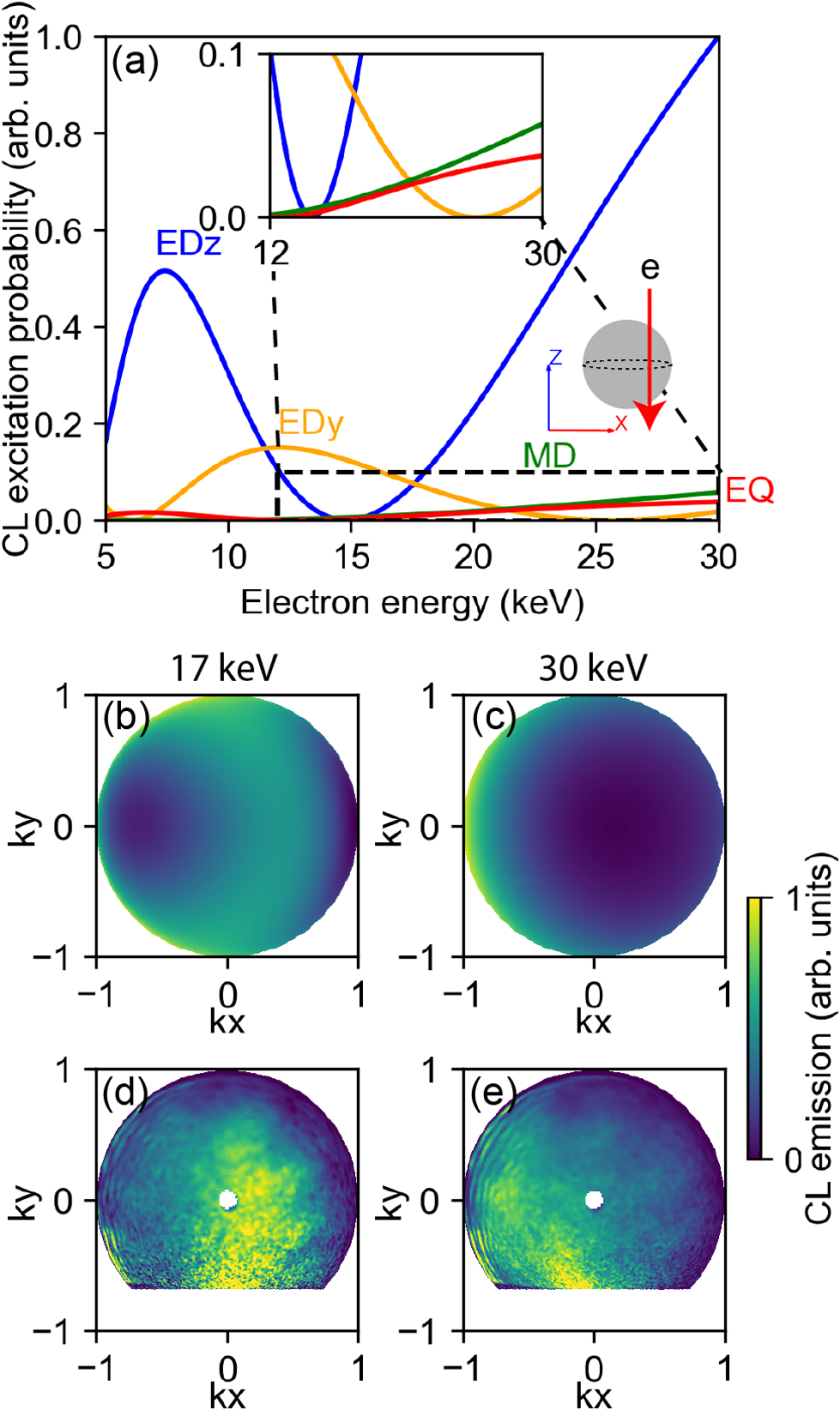
Analysis of directional CL emission toward the upper hemisphere from a Si nanosphere with *r* = 85 nm excited with an electron beam with energy ranging from 5 to 30 keV with *b* = 42 nm. (a) CL excitation probability between electron and ED*_z_* (blue), ED*_x_* (orange), MD (green), and EQ (red) modes versus electron energy, with the low CL intensity modes shown in the inset. (b, c) Calculated and (d, e) measured far-field radiation intensities in the positive *z*-direction (where the electron comes from), excited with a (b, d) 17 keV and (c, e) 30 keV electron beam. The experimental data are obtained with a *λ* = (600 ± 25) nm bandpass filter, and the theoretical data are calculated at *λ* = 585 nm.

To experimentally study the far-field radiation profiles, we perform angle-resolved CL (AR-CL) measurements at λ = (600 ± 25) nm for electron energies of 17 and 30 keV (Figure 5d,e). The data show clear right and left beaming for the two energies, respectively. This is in good agreement with the calculated radiation patterns in Figure 5b,c. At 17 keV the interference of the ED*_z_* and ED*_x_* modes in the far field creates strong directionality toward the impact position, while at 30 keV we see a stronger contribution of the ED_*z*_ mode superimposed on contributions of MD and EQ modes that make the radiation pattern asymmetric. Consequently, selective mode coupling can be used to generate and tune CL emission with strong directionality by controlling the electron beam energy.

Another important parameter to tune the directionality of CL emission is the impact parameter *b*, which we investigate for the same Si nanosphere at a fixed electron beam energy of 30 keV. Figure 6a shows the coupling efficiency of the electron beam with the EQ, ED*_z_*, ED*_x_*, and MD modes as a function of impact parameter *b*. The inset shows a magnified view of the lower-intensity modes. We clearly observe two interaction regimes: close to the nanosphere’s center (*b* = 20 nm) the ED*_z_* mode contributes strongest, while closer to the edge the electron can couple to all modes. For two exemplary impact parameters (*b* = 42 nm and *b* = 76 nm) we calculate the AR-CL emission, shown in Figure 6b,c, respectively. In both cases we notice a strong directionality toward the negative *k*_*x*_-direction, i.e., opposite to the electron beam excitation. However, for excitation closer to the edge (*b* = 76 nm), we also find a contribution of the ED*_x_* mode which radiates into the upper hemisphere, as well as radiation from the ED*_z_* + MD modes in the horizontal (in-plane) direction.

**Figure 6:**
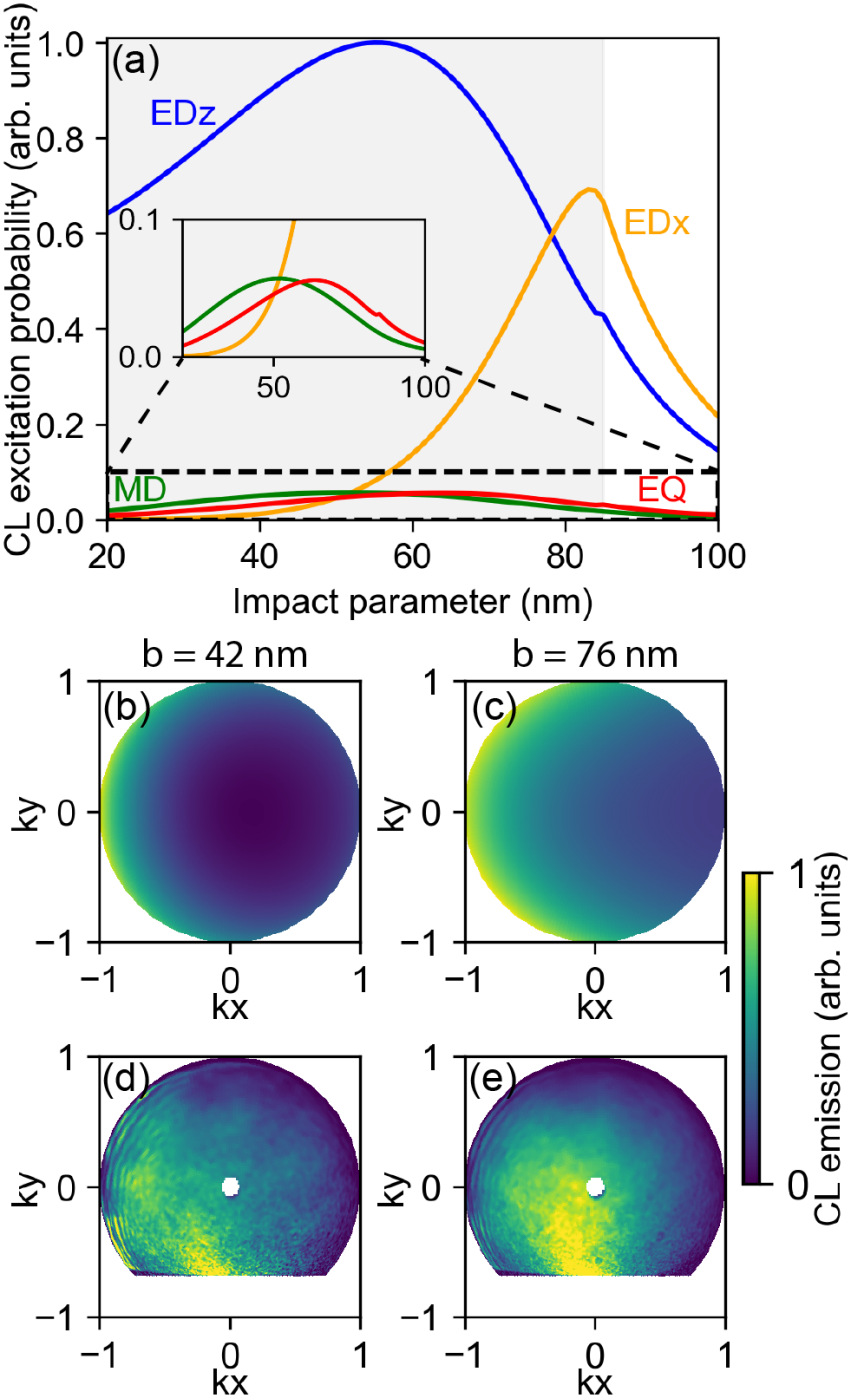
Measured and calculated effect of the impact parameter *b* on the directionality of the CL emission toward the upper hemisphere from a Si nanosphere with *r* = 85 nm excited with a 30 keV electron. (a) CL excitation probability between electron and ED*_z_* (blue), ED*_x_* (orange), and MD (green) versus *b*, the gray background shows the inside and the outside of the particle. The inset shows the magnified *y*-axis for low CL intensity modes. Calculated (b, c) and measured (d, e) far-field radiation intensities in the positive *z*-direction (where the electron comes from), for excitation at (b, d) *b* = 42 nm, and (c, e) *b* = 76 nm. The experimental data is obtained with a *λ* = (600 ± 25) nm bandpass filter, and the theoretical calculation is done at *λ* = 585 nm.

Similar trends are also seen in the experiments represented in Figure 6d,e, where we find a strong horizontal (in-plane) directionality for *b* = 42 nm, and an additional radiation component into the upper hemisphere for excitation at *b* = 76 nm. While experiments and theory generally agree well, we also notice in comparing Figure 6c,e that the relative contribution of MD + EDz (horizontal directionality) and MD + ED*_x_* (vertical directionality) to the overall directionality do not fully match. The experimental data indicate that the contribution of the ED*_x_* mode is stronger than suggested by the calculations. We attribute this deviation to the effect of the substrate: the 15 nm-thin Si_3_N_4_ substrate changes the induced electric field in the nanosphere and thereby changes the relative electron-coupling strength to the ED*_z_* and the ED*_x_* mode.

## Conclusions

In conclusion, we experimentally and theoretically studied the effect of the electron beam energy and impact parameter on the cathodoluminescence (CL) emission of crystalline Si nanospheres. In the experiments, we spectrally identified electric and magnetic dipolar and quadrupolar Mie resonances in the Si nanospheres upon electron beam excitation, in good agreement with theory. Two-dimensional spectral CL excitation maps show the modal excitation profiles at high spatial resolution, only limited by the electron beam spot size and small beam straggle.

The spectral and spatial distributions are in good agreement with a model in which the electron resonantly couples to specific modal field distributions with characteristic spatial frequencies that are determined by the electron velocity and resonance wavelength. The accessible electron energies in the SEM offer a wide range of velocities and thus probe a wide span of spatial frequencies, enabling the studies presented here. We use the selective modal excitation mechanism to control the directionality of the CL emission. Both electron energy and impact parameter are used to selectively excite resonant modes that interfere in the far field to create well-defined angular beaming.

The insights in this paper can inspire novel designs of lasers, light-emitting diodes, sensors, and photovoltaics, utilizing the coupling of nanosized optical emitters to resonant nanostructures. The resonant nanostructures can function as effective antennas by directing light generated at the nanoscale to the far field. With the resonant electron-mode coupling mechanism solidly confirmed in these experiments it becomes possible to design this coupling for a wide range of electron-light-matter interactions, in further advanced CL, EELS and PINEM spectroscopies.

## Methods

### Analytical Models

In our initial electron-to-mode coupling model the CL emission is taken proportional to the energy lost by the electron as it passes through the electric field of a specific single mode. The CL emission (Γ_CL_) for an impact parameter (***R*** = (*x*,*y*)), and frequency (ω) can be calculated as the path integral along the electron trajectory over the z-component of the electric field of a specific mode (*E*_*z*_), given by

3 $$\Gamma_{\mathrm{CL}}\left(R,\omega \right)\propto {\left|\int dz\;E_{z}\left(R,z\right)e^{-i\left(\omega /v\right)z}\right|}^{2}$$

with *v* the electron speed. With this method, valid in the nonrecoil approximation and when considering the modes individually, we can study the coupling of the electron to single modes. We calculate the induced electric field for a single mode using Mie theory. By using vector spherical harmonics, we calculate the induced electric fields for different modes, excited by a plane wave.^23^ While this model presents an intuitive way to determine the coupling to different modes, it does not consider how these modes couple with each other.

For the full solution, we use a Mie-based solution of the CL emission probability.^25^ It enables us to consider multiple modes at the same time and is then crucial for the spectral and directionality study. The model gives the expansion of the electric and magnetic fields induced by an electron crossing a sphere in vector spherical harmonics, where the total electric (**E** ^**II**^ (***r***,ω)) and magnetic fields ( $H^{II}\left(r,\omega \right)$ ) outside the particle (region II) are given by

4 $$\begin{array}{l} E^{II}\left(r,\omega \right)=\sum_{l=1}^{\infty }\sum_{m=-l}^{+l}\left\{b_{lm}^{II}h_{l}^{+}\left(k_{0}r\right)X_{lm}\left(\theta ,\phi \right)+\frac{i}{k_{0}}a_{lm}^{II}\nabla \times h_{l}^{+}\left(k_{0}r\right)X_{lm}\left(\theta ,\phi \right)\right\}\\ H^{II}\left(r,\omega \right)=\frac{1}{Z_{0}}\sum_{l=1}^{\infty }\sum_{m=-l}^{+l}\left\{a_{lm}^{II}h_{l}^{+}\left(k_{0}r\right)X_{lm}\left(\theta ,\phi \right)-\frac{i}{k_{0}}b_{lm}^{II}\nabla \times h_{l}^{+}\left(k_{0}r\right)X_{lm}\left(\theta ,\phi \right)\right\} \end{array}$$

Here $b_{lm}^{\mathrm{II}}$ and $a_{lm}^{\mathrm{II}}$ are the expansion coefficients of the scattered field for mode l>0 and orientation (−l≤m≤l) with the analytic formulas from ref 25, $X_{lm}$ are the vector spherical harmonics evaluated in spherical coordinates, and $h_{l}^{+}$ the spherical Hankel function of the first kind. The CL emission radiated at a point ***r*** = (*R*, θ, ϕ) in spherical coordinates is proportional to the Poynting vector given by

5 ΓCL(ω,r)∝R2πℏωRe{EII(r,ω)×HII*(r,ω)}·r̂

In order to compute the CL spectrum, we integrate the Poynting vector over all angles and sum over the modes (l) and orientations (*m*). This results in the expression

6 $$\Gamma_{\mathrm{CL}}\left(\omega \right)=\frac{1}{\pi \hbar \omega Z_{0}k_{0}^{2}}\sum_{l=1}^{\infty }\sum_{m=-l}^{+l}\left\{{\left|b_{lm}^{\mathrm{II}}\right|}^{2}+{\left|a_{lm}^{\mathrm{II}}\right|}^{2}\right\}$$

with *Z*_0_ the impedance in free space $\sqrt{\frac{\mu_{0}}{\varepsilon_{0}}}$ . By selecting the modes of interest (l,m) , either magnetic or electric in eqs 4 and 6, we isolate the contribution of each multipole to the CL radiation.

### Cathodoluminescence Measurements

The measurements were performed in a FEI Helios 600, ThermoFisher Scientific, Inc. SEM equipped with a half-parabolic mirror and spectral and angular CL acquisition SPARC system from Delmic B.V. The acquisition of the CL maps was performed using a pixel size of 14 nm and an exposure time of 1 s. All spectra and maps shown in this paper were taken from the same nanosphere, and the CL background signal from the supporting Si_3_N_4_ membrane was subtracted. The systems’ response was calibrated using transition radiation from a single crystalline aluminum sample as described in literature^32^ and this has been used to correct all experimental CL data. The study on directional emission was done at a smaller Si nanosphere of a radius of 85 nm. For the angle-resolved CL emission measurements, the grating was removed from the optical path, and the reflected light from the mirror is directed onto a 2D CCD camera to measure in Fourier mode. For these measurements bandwidth filters with a bandwidth of 50 nm, and an exposure time of 5 s were used. In both spectral and angular CL measurements, the electron beam current was 1.4 nA.

### Fabrication of the Sample

The Si nanospheres were fabricated starting by crushing SiO lumps to powder and annealing it in N_2_ atmosphere. The powder was etched in a hydrofluoric acid (HF) to remove the SiO_2_ matrices. Finally, the freestanding Si nanospheres were transferred to methanol and then ultrasonicated and filtered to particle sizes around 100 nm radius.^26^ Next, we drop casted 5 μL from the suspension of solution onto a 15 nm-thin Si_3_N_4_ support film for TEM provided by PELCO and dried it. By postprocessing the SE images, we determined the particle radii of 96 and 85 nm for the spectral and angular CL analysis, respectively.

## Supplementary Material



## Notes

Measured and calculated electron-energy dependent CL spectra (Figure S1) for a silicon nanosphere with a radius of 96 nm excited at the edge and the center; the CL emission line profiles along the center of this particle (Figure S2); Monte Carlo simulations of electrons passing through silicon for an electron energy of 15 and 30 keV (Figure S3); and the calculated CL spectrum for a 96 nm radius particle excited at *b* = 86 nm with a 30 keV electron beam decomposed into the contributions of the MD, ED, MQ, and EQ (Figure S4) (PDF)
