# Supplementary material for: Electron-Energy Dependent Excitation and Directional Far-Field Radiation of Resonant Mie Modes in Single Si Nanospheres
Source: ACS Photonics. 2025 Jul 24;12(8):4161–70. doi: 10.1021/acsphotonics.5c00173 (PMC12372165; doi:10.1021/acsphotonics.5c00173)
Supplement: Supplementary file 1 [file ph5c00173_si_001.pdf]

Electron-energy dependent excitation and directional far-field radiation of resonant Mie modes in single Si nanospheres

Théo Soler[1,a], Evelijn Akerboom[1,a,*], P. Elli Stamatopoulou[b], Hiroshi Sugimoto[c], Minoru Fujii[c], Saskia Fiedler[a], and Albert Polman[a,*]

[a] *Center for Nanophotonics, NWO-Institute AMOLF, Science Park 104, 1098 XG Amsterdam, the Netherlands*

[b] *Institute of Nanotechnology, Karlsruhe Institute of Technology, Kaiserstr. 12, 76131 Karlsruhe, Germany*

[c] *Department of Electrical and Electronic Engineering, Graduate School of Engineering, Kobe University, Rokkodai, Nada, Kobe 657-8501, Japan*

[*]Email: e.akerboom@amolf.nl, a.polman@amolf.nl

[1]*T.S. and E.A. contributed equally to this work*

S1: Electron energy dependent CL – spectra for a Si particle

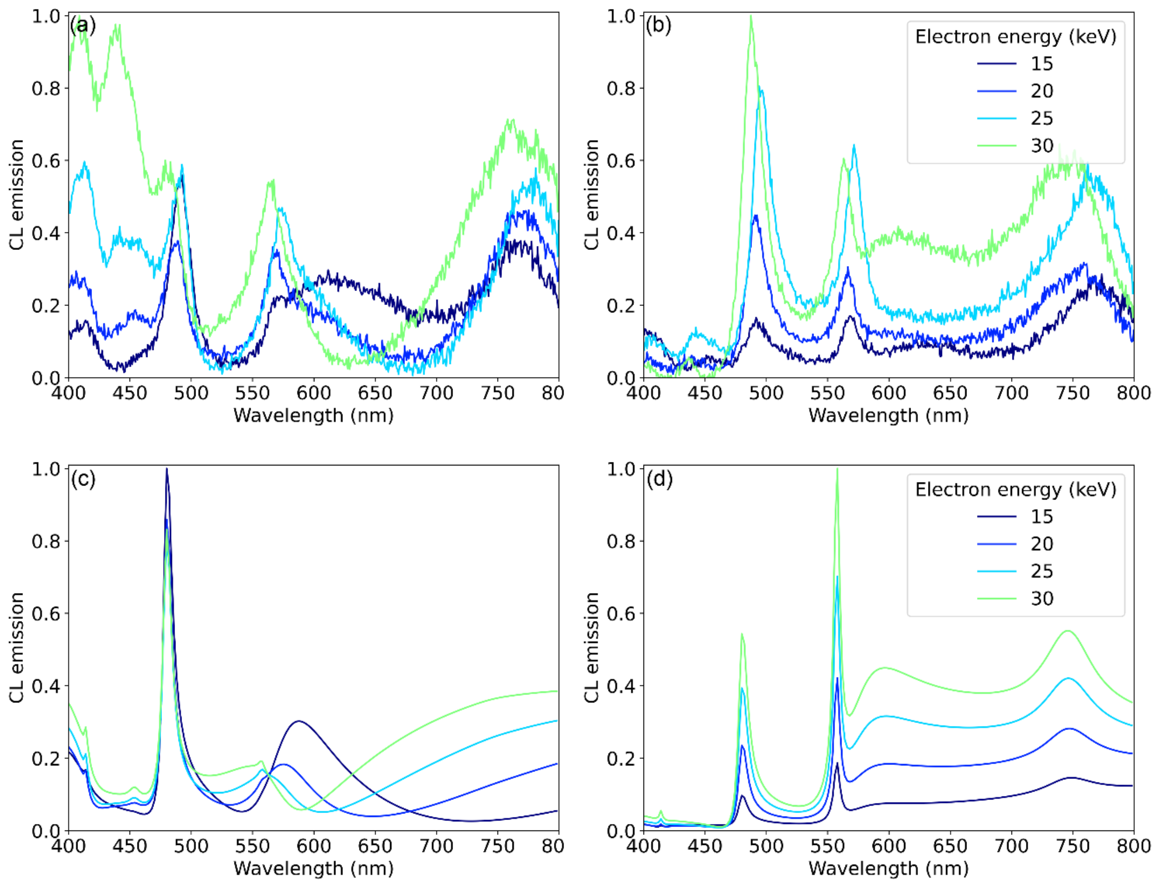

Figure S1. (a, b) Measured and (c, d) calculated CL spectra for a Si nanosphere with a radius of 96 nm, excited by a 15, 20, 25, and 30-keV electron beam with (a, c) center-excitation and (b, d) at b = 86 nm.

S2: Electron energy dependent CL emission – line scan over a Si nanosphere

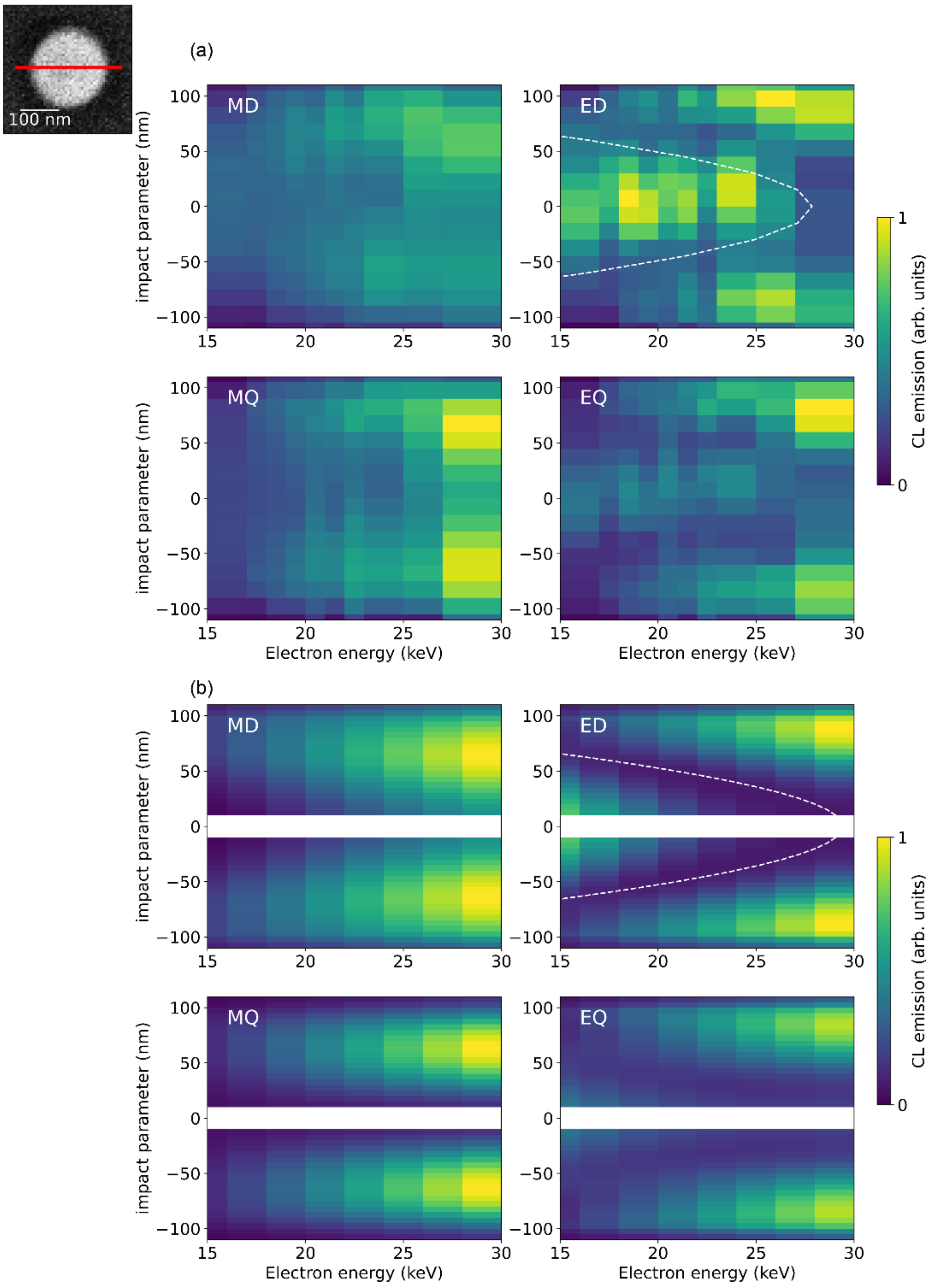

Figure S2. (a) Measured and (b) calculated CL line profile along the red line shown in the inset SE image. The line profiles are shown for a 15-30 keV electron beam exciting the same Si nanosphere (r=96 nm) as shown in Figures 2 and 3 in the main text, with the impact parameter varying from -100 nm to 100 nm. Experimental data are averaged over a spectral bandwidth of 4 nm, with a centre wavelength of 487 nm (EQ), 563 nm (MQ), 605 nm (ED), and 744 nm (MD). The theoretical CL maps are calculated for wavelengths of 450 nm, 557 nm, 595 nm, and 745 nm, respectively. The white dashed line shows the rule-of-thumb for the expected minima of the phase-matching condition for the ED, $q \sim 2n\pi/D^*$, for n=2 and with D^* the height of the particle at a certain impact parameter (for a spherical particle with radius R, the height at a certain impact parameter is given by $D^* = 2\sqrt{R^2 - b^2}$).

Figure S2 shows the line profiles of the CL emission along the center of the nanosphere (see the SE image in the inset). The data are shown for a 15-30 keV electron beam exciting the same Si nanosphere

(r=93 nm) as shown in Figures 2 and 3 in the main text, with the impact parameter varying from -100 nm to 100 nm. The experimental data (Fig S2a) clearly shows the different profiles for specific modes: while the magnetic modes show a high intensity at the edge of the particle (b~75 nm) independent of electron energy, the electric modes show an electron energy dependent intensity. Especially in the minimum this is very clear: at 30 keV electron energy, the minimum appears in the center of the particle, around b = 30 nm, and this minimum moves more towards the edge for slower electrons. This is due to the phase-matching condition. The rule of thumb for an electric dipole mode follows that the maxima of CL emission are found at $q \sim(2n+1)\pi/D$, and the minima at q ~$2\pi n/D$. In this case this follows the minima nicely; in Figure S2b the rule of thumb for the second order CL minima (q ~$4\pi/D$) is overlaid with the line profile in dashed white and it follows the minima perfectly. Effectively, electron-energy dependent CL measurements allow for the determination of the thickness of the particle at different impact parameters. This is less clear for the MD, MQ, and EQ mode, due to their less distinctive electric near-field distribution: unlike the other modes, the ED has high E_z components at the surface of the nanosphere (as visible in Figure 1b).

S3: Monte-Carlo simulations for electrons traversing a planar Si surface

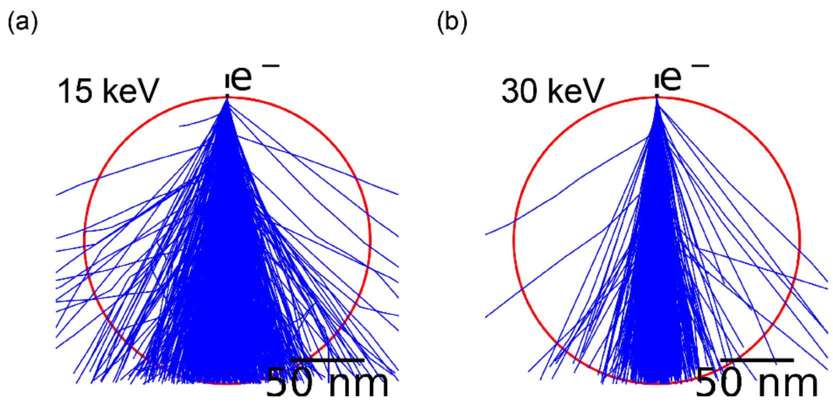

Figure S3. Monte-Carlo statistics of the penetration depth (z_{max}) of (a) 15 keV, and (b) 30 keV electrons penetrating a planar Si surface. The insets show the dispersion of electron trajectories during propagation through the Si. Contours of a Si particle with a radius of 100 nm are shown as a guide to the eye.

S4: Calculated CL spectra decomposed in the different modes

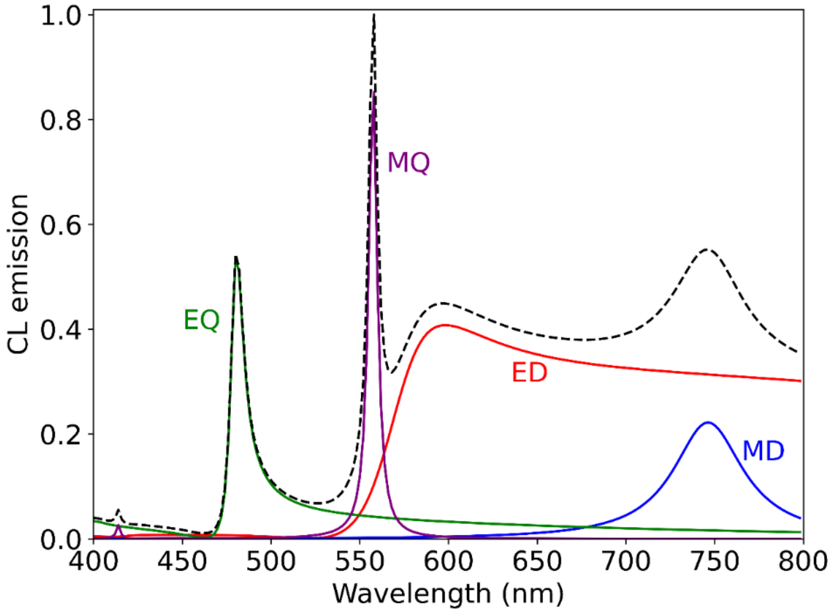

Figure S4. Calculated CL spectra for a 30 keV electron beam exciting a silicon nanosphere with a radius of 96 nm at an impact parameter of 86 nm. The CL spectra are decomposed in the contribution of the different modes, and in black dashed the total spectrum with ℓ_{max}= 2.
